# Deep Subsurface Life from North Pond: Enrichment, Isolation, Characterization and Genomes of Heterotrophic Bacteria

**DOI:** 10.3389/fmicb.2016.00678

**Published:** 2016-05-10

**Authors:** Joseph A. Russell, Rosa León-Zayas, Kelly Wrighton, Jennifer F. Biddle

**Affiliations:** ^1^College of Earth, Ocean and Environment, University of DelawareLewes, DE, USA; ^2^Department of Microbiology, The Ohio State UniversityColumbus, OH, USA

**Keywords:** deep biosphere, bacteria, cultivation, genome, amplicon

## Abstract

Studies of subsurface microorganisms have yielded few environmentally relevant isolates for laboratory studies. In order to address this lack of cultivated microorganisms, we initiated several enrichments on sediment and underlying basalt samples from North Pond, a sediment basin ringed by basalt outcrops underlying an oligotrophic water-column west of the Mid-Atlantic Ridge at 22°N. In contrast to anoxic enrichments, growth was observed in aerobic, heterotrophic enrichments from sediment of IODP Hole U1382B at 4 and 68 m below seafloor (mbsf). These sediment depths, respectively, correspond to the fringes of oxygen penetration from overlying seawater in the top of the sediment column and upward migration of oxygen from oxic seawater from the basalt aquifer below the sediment. Here we report the enrichment, isolation, initial characterization and genomes of three isolated aerobic heterotrophs from North Pond sediments; an *Arthrobacter* species from 4 mbsf, and *Paracoccus* and *Pseudomonas* species from 68 mbsf. These cultivated bacteria are represented in the amplicon 16S rRNA gene libraries created from whole sediments, albeit at low (up to 2%) relative abundance. We provide genomic evidence from our isolates demonstrating that the *Arthrobacter* and *Pseudomonas* isolates have the potential to respire nitrate and oxygen, though dissimilatory nitrate reduction could not be confirmed in laboratory cultures. The cultures from this study represent members of abundant phyla, as determined by amplicon sequencing of environmental DNA extracts, and allow for further studies into geochemical factors impacting life in the deep subsurface.

## Introduction

The marine environment harbors numerous uncultivated microbes with estimates of culturable marine bacteria around 0.01 to 0.1% of cells present (Kogure et al., 1979; Connon and Giovannoni, 2002). The “great plate count anomaly” (Staley and Konopka, 1985) extends into the marine deep biosphere, yet there is still a need for cultivation-based research to better fill critical knowledge gaps, transforming our understanding of the biogeochemical impacts in the deep biosphere (Parkes et al., 2009; Fichtel et al., 2012; Lomstein et al., 2012; Lever et al., 2013; Ciobanu et al., 2014). Cultivated microbes from the deep marine subsurface have often come from anoxic sediments, including anaerobes such as *Desulfovibrio profundus* (Bale et al., 1997) and *Methanoculleus submarinus* (Mikucki et al., 2003). Facultative anaerobes (*Shewanella profunda*, Toffin et al., 2004) and numerous aerobes have been retrieved from anaerobic sediments (D’Hondt et al., 2004; Biddle et al., 2005). Overall, most isolates from the deep biosphere have poor environmental context to date, and few isolated aerobes have originated from aerobic sediments.

**FIGURE 1:**
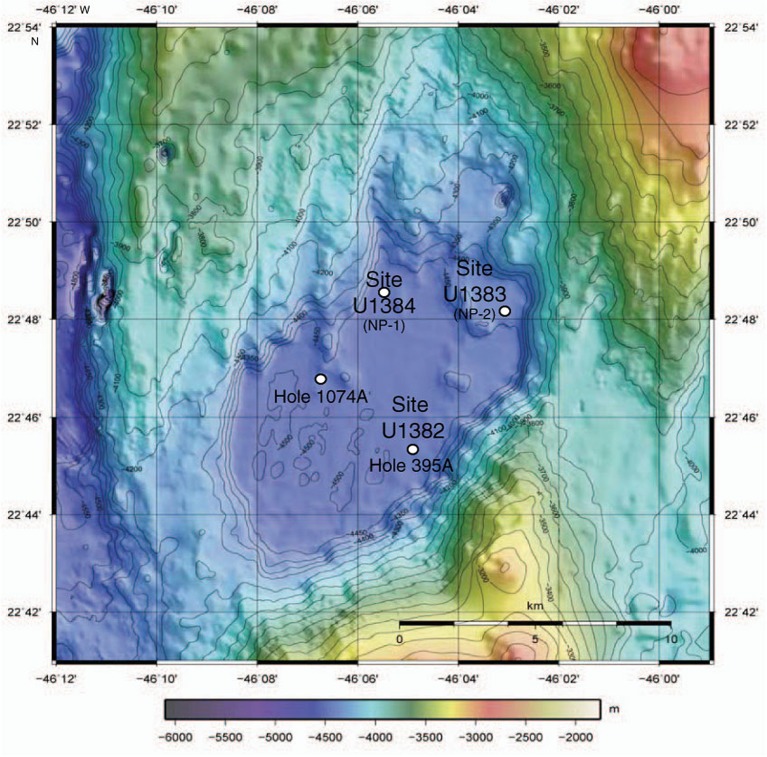
**Bathymetric map of North Pond in the Atlantic Ocean, showing drill sites (from Expedition 336 Scientists, 2012).** Isolates in this paper are from Site U1382, Hole U1382B and enrichments were also started from Hole U1382A, Site U1383 (Holes U1383C and U1383E) and Site U1384 (Hole 1384A; Supplementary Table S1). Sites from IODP Expedition 336 are shown (Site #, NP #), as are holes previously drilled in the area (DSDP Hole 395A, ODP Hole 1074A).

The collection of deep biosphere sediment and basalt via drilling during International Ocean Drilling Program (IODP) Expedition 336 to North Pond offered an opportunity to initiate cultivation studies across several subsurface environments: young aerobic sediments, older anoxic sediments, still older aerobic sediments, and 7-million-year-old ridge flank basalt (Expedition 336 Scientists, 2012). North Pond is a sediment-filled basin between exposed basalt outcrops, where temperature differences exist between warmer out-crops closer to tectonic activity at the Mid-Atlantic Ridge and colder outcrops further away at the southwest section of the basin (Langseth et al., 1992; Edwards et al., 2012). In the colder, southwest corner of the basin, Site U1382 was drilled to a depth of 210 m below seafloor (mbsf), including 90 m of sediment from Hole U1382B and 120 m of basement from Hole U1382A (Expedition 336 Scientists, 2012; **Figure 1**).

At Site U1382, oxygen penetrated the sediment to approximately 4 mbsf and returned at approximately 68 mbsf due to flow from the underlying basalt (Orcutt et al., 2013). In addition to oxygen, other electron acceptors such as nitrate are reported (Edwards et al., 2012; Wankel et al., 2015). While electron acceptor concentrations are similar at these depths, the buried organic carbon at 68 mbsf is over 6 million years older than at 4 mbsf and has presumably undergone some level of remineralization given that oxygen is used up in the middle of the sediment column (Burdige, 2007). Currently, our knowledge of oxygen- and nitrogen-cycling heterotrophy in North Pond sediments are based on modeling of nitrogen isotopes and the examination of sediment slurries with labeled carbon substrates (Picard and Ferdelman, 2011; Wankel et al., 2015). Physical characteristics of the sediment that enhance the delivery of nutrient/electron-acceptor rich pore-water are more important than indigenous carbon substrates for constraining heterotrophy (Picard and Ferdelman, 2011). Active nitrogen cycling in North Pond sediments does not follow canonical patterns of redox separation between consumption and regeneration of nitrate, with overlapping zones of aerobic (nitrification) and anaerobic (denitrification) processes (Wankel et al., 2015). North Pond sediments are likely to be representative of a majority of the global seafloor, as oligotrophic water columns such as those overlying North Pond comprise a majority of the global ocean (D’Hondt et al., 2009).

Here we report the enrichment, isolation, initial characterizations and genomes of three isolated aerobic heterotrophs from North Pond sediments; an *Arthrobacter* species from 4 mbsf, and *Paracoccus* and *Pseudomonas* species from 68 mbsf. Dissimilatory nitrate reduction could not be induced under laboratory conditions of all three isolates, however, genomic data suggests a metabolic capability for denitrification in the *Arthrobacter* isolate from 4 mbsf and *Pseudomonas* isolate from 68 mbsf at U1382B, depths where we expect this metabolism to be occurring (Wankel et al., 2015). Based on amplicon sequencing abundances, these isolates are rare members of abundant phyla in the environment. To our knowledge, these are the first aerobic isolates reported from oxygenated deep marine sediments.

## Materials and Methods

### Sampling

Samples were acquired from the scientific drilling vessel *JOIDES Resolution* during IODP Expedition 336 in November, 2011 (**Figure 1**). Samples were taken from Holes U1382A, U1382B, U1383C, U1383E, and U1384A (Supplementary Table S1). Whole round core sections were sampled at 10 m intervals when available. Samples for DNA extraction were immediately stored at -80°C and kept at this temperature for the remainder of the cruise and during shipping to shore-based laboratory. Samples for cultivation were stored at 4°C for no longer than 2 h before being inoculated into enrichment media. Enrichment media was prepared from literature-sourced recipes to attempt to cultivate aerobic heterotrophs (GYPS; Burgaud et al., 2009), sulfate reducers (Pfennig et al., 1981), methanogens (Wolfe et al., 2011), sulfide oxiders (Nelson et al., 1986), iron reducers (Wrighton et al., 2011) iron oxidizers/nitrate reducers (Straub and Buchholz-Cleven, 1998), Mn oxiders (Krumbein and Altmann, 1973), and Mn reducers (Burdige and Nealson, 1985) from both sediments and basalts. Numerous enrichments were prepared across different samples and depths with negative controls of uninoculated broth (Supplementary Table S1). However, confirmed growth was only observed in the aerobic heterotroph enrichments from sediments taken by Advanced Piston Coring (APC) at Site U1382B at 22°45.353′N, 46°04.891′W; as such, this is the only enrichment we will report in detail.

### Culture Media, Growth Conditions, and Growth Measurements

Original GYPS media for aerobic heterotrophs was prepared in 1 L batches onboard the *JOIDES Resolution* (JR) and consisted of the following; 1 g each of glucose, peptone, starch, and yeast extract, 1 L filtered/autoclaved seawater (Burgaud et al., 2009). Media was then autoclaved for 1 h. After autoclaving and allowing 30 min for cooling, 5 mL Wolfe’s vitamins and 5 mL Wolfe’s minerals was added and 40 mL was aseptically dispensed in autoclaved 60 ml glass vials and capped with rubber septa, which were removed during sample addition and every week after to reoxygenate the media. This dispensing, and all sediment additions, were performed in the sterile flow hood aboard the JR. The top 3–5 mm of sediment from each whole round core section was removed with a flame-sterilized spatula to reduce any potential drilling contamination and 3 cubic centimeter samples were taken from the center of the core using sterilized cut-off syringes. Care was taken to sample from the *interior* of the core to avoid sampling sediment contaminated with seawater during the drilling process. This drilling-induced seawater contamination was monitored using the fluorescent microsphere method, as described previously (Smith et al., 2000). Microsphere data for sediment horizons from which isolates derived is <1 microsphere per field of view for interior core samples, indicating a very low risk that isolates originated from seawater (Expedition 336 Scientists, 2012). Basalt whole-round core samples were broken into smaller pieces with a flame-sterilized chisel in a flame-sterilized steel rock processing box. After processing, 2–3 g of basalt chips were used as inoculum in enrichment media. The sediment and basalt subsamples were immediately inoculated into enrichment vials aerobically and the vials were capped with rubber septa and stored at 4°C. Growth was observed by turbidity of media from sediment samples from 4 and 68 mbsf (section 1 and 8H) within 2 days and transfers into new media were done every 5 days for the remainder of the cruise. At the end of the cruise, culture vessels were crimp-sealed for shipping to the Biddle Lab at 4°C. Once on shore, enriched heterotrophs were plated on Difco Marine Agar 2216 plates and placed at 10°C under aerobic conditions. Individual colonies were picked and streaked on new plates in five successive rounds of isolation at 10°C. Culture media for anaerobic growth on ${NO}_{3}^{-}$ was composed of the following, in 500 mL dH_2_O; 0.75 g of KH_2_PO_4_, 0.15 g of NH_4_Cl, 1 g of KNO_3_, 5 mM (NH_4_)_2_Fe(SO_4_)_2_^.^6H_2_O, 0.5 mg resazurin, 4% w/v Sigma sea salts, 10 mM glucose, 1 g peptone (Samuelsson, 1985). Culture media for Mn(II) oxidation was prepared as described previously (Krumbein and Altmann, 1973), in 1 L; 75% natural seawater, 25% dH_2_O, 20 mM HEPES, 2 g peptone, 0.5 g yeast extract, 1 mM MnCl_2_ (Templeton et al., 2005). Transfers were performed by growing isolates to late log phase in Difco Marine Broth 2216C at 10°C and 100 μl of this culture was used as inoculum to 40 mL of ${NO}_{3}^{-}$ and 40 mL of Mn(II) oxidizing media (Krumbein and Altmann, 1973) in 60 ml glass vials, capped with rubber septa. Cultivation on nitrate and manganese media was attempted three times for each isolate. Growth temperature tests were initially performed at 4, 12, 22, 37, 42, and 50°C on solid Difco Marine 2216C agar media. Due to equipment limitations on shaking incubators, we only measured growth rates at temperatures starting at 12°C. For growth rate measurements, cells were grown in liquid Difco Marine Broth with shaking and 500 μl timepoints were taken to measure optical density in reference to uninoculated media at 600 nm wavelength on a Nanodrop 2000c (ThermoFisher Scientific). Glycerol stocks of each isolate were prepared and frozen at -80°C for long term archiving. They are available to researchers upon request.

### DNA Extractions and Sequencing

Isolates were grown to late stationary phase in 5 ml of media. Cultures were centrifuged for 10 min at 4000 rpm to generate a cell pellet. The cell pellet for each isolate was re-suspended in 500 μl dH_2_O and DNA was extracted using the UltraClean Microbial DNA Isolation Kit from MoBIO Laboratories, Inc. (Carlsbad, CA, USA). Full-length 16S rRNA amplicons were generated with bacterial primers 8F/1492R (Eden et al., 1991) with the following PCR protocol; 94°C for 2 min, (94°C for 30 s, 59°C for 1 min, 68°C for 2 min) × 30 cycles, 68°C for 5 min, then cloned with the TOPO TA Cloning Kit for Sequencing (Life Technologies Inc., Grand Island, NY, USA). Ten clones per isolate were picked and sent for Sanger sequencing at GeneWiz Inc., (South Plainfield, NJ, USA) to confirm culture purity. Full length 16S rRNA DNAs are deposited under NCBI accession numbers KU707917–KU707919. Genomic DNA was sent to the United States Department of Energy’s Joint Genome Institute for full genome sequencing. Isolate genomes and raw sequencing data are available from the GOLD database via accession numbers: *Pseudomonas stutzeri* NP_8HT: Gp0114906, *Arthrobacter subterraneus* NP_1H: Gp0115197, *Paracoccus* sp. NP_8HO: Gp0114905 and under NCBI project ID: 303658. For total microbial community analysis at both depths (4 and 68 mbsf), 10 g of sediment from each depth was processed with the PowerMax Soil DNA Isolation Kit from MoBio Laboratories, Inc. (Carlsbad, CA, USA). Total bacterial community was amplified from 2 ng template DNA using 16S rRNA primers 27F/338R via PCR protocol: 95°C for 2 min, (95°C for 30 s, 60°C for 1 min, 72°C for 2 min) × 35 cycles, 72°C for 5 min. An extraction blank was processed with the samples. All PCR products, including the one from the extraction blank, were sent for Illumina library prep and sequencing at Next Generation Sequencing Services (Shallowater, TX, USA). This involved a nested PCR approach, resulting in a minimal number of cycles occurring after initial PCR amplification, which may slightly reduce the diversity of the environmental samples. Amplicon sequencing data is available under NCBI accession numbers SRS1277883–SRS1277884.

### Sequence Data Processing and Analysis

The raw data from 16S rRNA gene amplicon Illumina reads were converted to a fastq file in QIIME version 1.8 (Caporaso et al., 2010) with the *convert_fastaqual_fastq.py* script. Primers and barcodes were extracted with the *extract_barcodes.py* script and sequences were de-multiplexed via *split_libraries_fastq.py* and an in-house perl script. 1,846 operational taxonomic unit (OTUs) were shared between the extraction blank and the section 1H sample. 1,446 OTUs were shared between the extraction blank and the section 8H sample. The most abundant taxa associated with these OTUs were from families often observed as common kit contaminants, such as *Propionibacteriaceae, Enterobacteriaceae, Ralstoniaceae*, and *Bradyrhizobiaceae* (Salter et al., 2014). Several proteobacterial OTUs, including *Pseudomonas* and *Paracoccus* genera, were observed in the extraction blank. No *Arthrobacter*-related OTUs were observed in the blank. All OTUs shared between samples and the extraction blank were removed before further analysis. Sequences were then uploaded to the SILVA NGS pipeline^1^ (Quast et al., 2013) where default quality-trimming parameters were used, sequences were aligned with the SINA aligner (Pruesse et al., 2012), and OTU clustering was performed at 98% sequence identity. Taxonomy was called against the SILVA database (Quast et al., 2013). OTUs identified in the extraction blank sample that were also seen in sediment samples were removed from further analysis. The resulting OTU table was used for creation of taxonomy figures. Full-length 16S rRNA gene amplicon data was trimmed in Sequencher v.5.0.1 (GreenCodes Corp., Ann Arbor, MI, USA), sequences aligned with the online SINA aligner^2^ and manually refined within ARB (Ludwig et al., 2004). Phylogenetic analysis was performed using ARB via a neighbor-joining method with 1000 bootstrap replicates on the highest quality clone sequence from each isolate. Genomes were quality trimmed using Nesoni^3^. We then assembled these genomes using SPAdes 3.1.1 in multi cell mode (Bankevich et al., 2012). The – – careful flag was used to reduce the number of mismatches and short indwells and to run error correction in the assembly. The assembly iteratively used kmers 21, 33, 55, and 77. PHRED quality offset for the input reads was set at 33. During assembly and initial annotation, we found that the raw data for the *Pseudomonas* isolate also included the *Paracoccus* genome. Therefore, after assembly by SPAdes, we separated the genomes using MaxBin (Wu et al., 2014), evaluated bin validity with VizBin (Laczny et al., 2015) and CheckM (Parks et al., 2015) and deposited the new genomes in the integrated microbial genomes (IMG) database (Markowitz et al., 2012; Accession numbers 74356, 79457, and 79456; **Table 1**). Genomes were annotated by Prokka (Seeman, 2014). Genes for nitrogen cycling were extracted from the Prokka output and annotations were double checked via manual BLAST analysis against the NCBI nr database^4^ and the Interpro protein domain database^5^. Additional genes found via IMG annotation were also added. Proteins annotated as active subunits of significant enzymes were aligned with close relatives of the nr database via MUSCLE (Edgar, 2004) and visually inspected and trimmed with Jalview software (Waterhouse et al., 2009). Trees were made using maximum likelihood in FastTree (Price et al., 2009). Carbohydrate active enzymes were identified by comparison with the Carbohydrate Active Enzymes database^6^ (Cantarel et al., 2009) via pFAM scan (Mistry et al., 2007).

**Table 1:** Genome statistics of three heterotrophic aerobic isolates from sediment of Hole U1382B collected during IODP Expedition 336.

Organism	Sediment depth (mbsf)	Genome size (Mb)	Contigs^∗^	Number of predicted genes	%GC	% completeness^∗^
*Arthrobacter* sp. NP1H	4	3.90	99	3674	65	99.1
*Pseudomonas* sp. NP8HT	68	4.74	24	4353	60	100.0
*Paraccocus sp.* NP8HO	68	3.32	21	3193	68	99.7

*^∗^Reported statistics are based on contigs >500 bp, completeness from CheckM calculation*.

## Results and Discussion

### Isolation and Phylogeny of Heterotrophs

**FIGURE 2:**
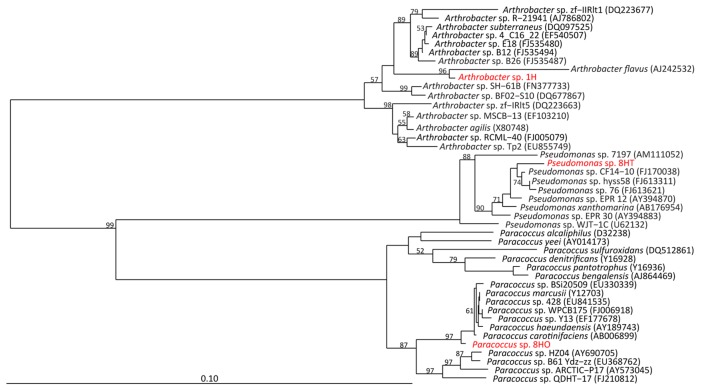
**Neighbor-joining tree of full length 16S rRNA gene sequences of isolates (red) to nearest database neighbors (black), based on 1000 bootstrap replicates.** Bootstrap values >50% are shown.

Only the heterotrophic enrichments yielded observable growth from these deep samples, despite the media being quite high in carbon compared to the *in situ* environment (Edwards et al., 2012). A single colony morphotype was observed on Difco marine agar media during successive rounds of isolation from the 4 mbsf sample (section 1H); a light tan color with moderate EPS production. Two separate morphologies were identified from the 68 mbsf sample (section 8H). The first colony morphotype was light tan colored with moderate EPS production. The second was a bright orange color colonies with low EPS production. Full-length 16S rRNA gene sequencing indicates the section 1H isolate as an *Arthrobacter* species from the *Micrococcaceae* within the *Actinobacteria*, the orange 8H isolate as a *Paracoccus* species from the *Rhodobacteraceae* within the *Alphaproteobacteria*, and the tan 8H isolate as a *Pseudomonas* species from within the *Gammaproteobacteria* (**Figure 2**). The isolates were all related to species found previously in the subsurface and sediments.

The section 1H *Arthrobacter* sp. isolate was closely related to *Arthrobacter* species retrieved from deep subsurface water in South Korea (*A. subterraneus*) and Himalayan ice (*Arthrobacter* sp. zf-IIRIt1), as well as marine sediment off Svalbard, Norway (*Arthrobacter* sp. SH-61B; **Figure 2**). The closest relative (96.7% 16S rRNA gene identity) is *A. flavus*, a psychrophile isolated from a cyanobacterial mat in a pond in the McMurdo Dry Valley, Antarctica (Reddy et al., 2000). This isolate is the most novel based on distance from known relatives, in terms of 16S rRNA identity of the three we cultivated. It had a typical rod/coccus morphology depending on growth stage, with diameters ranging from 0.9 to 2 μm.

Clone A45 from the orange 8H isolate branched close to *Paracoccus* species previously isolated from Korean salt marsh sediments (*Paracoccus* sp. HZ04), Chinese salt deposits (*Paracoccus* sp. Y13), and deep sediments of the Arctic Ocean (*Paracoccus* sp. BSi20509). It is most closely related (99.2% 16S rRNA gene identity) to *Paracoccus carotinifaciens*, an aerobic, orange-pigmented, Gram-negative, rod-shaped microbe originally isolated from soil (Tsubokura et al., 1999; **Figure 2**). Our isolate stained gram-negative and exhibited a primarily coccoid shape (average diameter of 0.85 μm) though some rod shapes were observed, indicating a potential for growth related morphological changes.

Clone A25 from the tan 8H isolate branched close to *Pseudomonas* species previously isolated from South China Sea sediments (*Pseudomonas* sp. CF14-10), sediments of the southwest Pacific Ocean (*Pseudomonas* sp. 76, *Pseudomonas* sp. hyss58), and its closest relative was isolated from East Pacific Rise hydrothermal sediments, *Pseudomonas* sp. EPR 12 (Vetriani et al., 2005; 99% 16S rRNA gene identity; **Figure 2**). The isolate was rod-shaped and approximately 0.9 to 1.5 μm long.

### Amplicon Sequencing from Enviromental Samples

**FIGURE 3:**
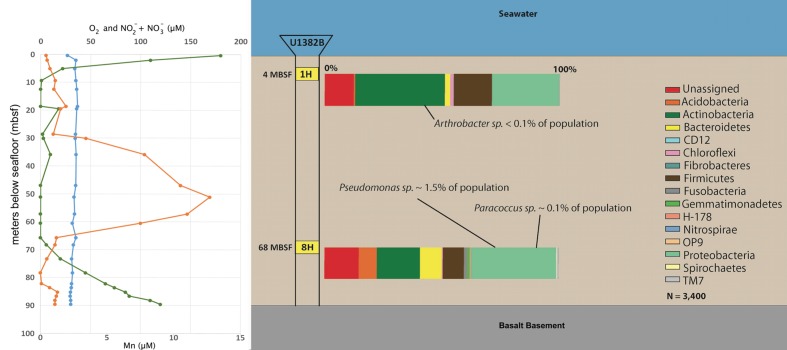
**Down-core O_2_ (green), Mn (orange), and NO_3_ + NO_2_ (blue) concentrations (from Expedition Expedition 336 Scientists, 2012 and Orcutt et al., 2013) and 16S rRNA gene taxonomic profile, by phyla, for bacteria in frozen sediments of sections 1H (4 mbsf) and 8H (68 mbsf).** Each amplicon sample was rarified to 3400 sequences for analysis. Notations regarding the percentage of isolate signatures are listed **(Table 2)**.

To determine relative abundance of our isolates in the context of the greater microbial community from which they were collected from, we generated 16S rRNA gene amplicon libraries from frozen samples of sections 1 and 8H (**Figure 3**). Our enrichments had generated isolates from the *Actinobacteria* in the shallow sample (1H) and *Proteobacteria* from the deeper sample (8H). Consistent with the enrichments, the *Actinobacteria* and *Proteobacteria* were the dominant phyla represented in 1 and 8H microbial communities. *Actinobacteria*, which includes *Arthrobacter*, were 36.7 and 14.8% of the population at 1 and 8H, respectively. *Proteobacteria* were 29.1 and 29.4% of the population at 1 and 8H, respectively. However, the isolates were in extremely low abundance with *Arthrobacter* sp. 1H comprising <0.1% of the population at section 1H (**Table 2**). At section 8H, *Paracoccus* sp. 8HO was 0.1% of the population and *Pseudomonas* sp. 8HT was 1.5% (**Figure 3**; **Table 2**). Since the primers used in this analysis were much less discriminatory than those used to verify the identity of the isolates (310 and 1484 bp PCR products, respectively, whereas full length 16S rRNA gene primers were used for isolate identification), and this involved a nesting priming approach, the abundances could increase if longer sequences were clustered for the environmental sample. Considering the relationship to other subsurface and sediment relatives that were also isolated, these organisms may not be numerous in these environments, but widely disbursed across these environments and respond well to cultivation. The same OTUs are found at different depths (**Table 2**), which correspond to different ages of sediment. Notably, the *Arthrobacter* is not found at 68 mbsf, which corresponds with the overall decrease of members of *Actinobacterium* phylum at depth (**Figure 3**). We presume that organisms are deposited at the surface of the sediment column, and that the *Actinobacter* may not survive the lowest oxygen portion of the sediment column as well as the *Proteobacteria*, which results in the abundance shift at depth (**Table 2**; **Figure 3**).

**Table 2:** Abundances of isolate small subunit ribosomal (rRNA) signatures *in situ*.

Depth (mbsf)	*Arthrobacter* isolate	*Pseudomonas* isolate	*Paracoccus* isolate
4	3 (0.001)	67 (0.025)	6 (0.002)
68	0	52 (0.02)	3 (0.001)

*First number is amount of sequences detected. Percent abundance is indicated in parentheses*.

### Cultivation Responses of Cultivated Bacteria

**FIGURE 4:**
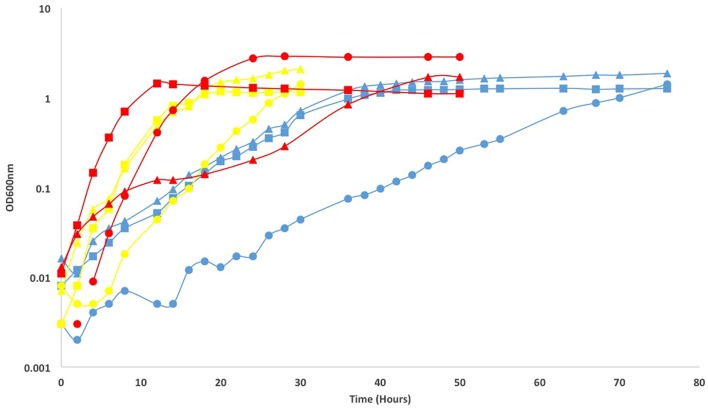
**Growth curves and calculated growth rates in parentheses (h^-1^) of North Pond isolates *Paracoccus* sp. 8HO (triangles), *Arthrobacter* sp. 1H (circles) and *Pseudomonas* sp. 9HT(squares) at 12°C (blue), 22°C (yellow), and 37°C (red).** *Paracoccus* sp. 8HO growth rates were 0.12, 0.24, and 0.06, per hour, at 12, 22, and 37°C, respectively. *Arthrobacter* sp. 1H growth rates were 0.06, 0.24, 0.36 and *Pseudomonas* sp. 8HT growth rates were 0.06, 0.24, and 0.48. Curves without a plateau, seen in *Paracoccus* sp. 8HO at 12°C may be caused by a build up in EPS material, not cell division, since cultures became sticky with increasing time, resulting in difficulty pipetting. This was not confirmed by direct cell counts.

The response of the isolates to varying temperatures (4, 12, 22, 37, 42, and 50°C) was measured on Difco marine broth agar, using organic carbon as an electron donor and O_2_ as an electron acceptor. For all three isolates, growth was very slow but detected at 4°C. No growth was detected at 50°C. Slow growth at 42°C was only seen for the *Pseudomonas* sp. and *Arthrobacter* sp.. Growth rates were measured for three temperatures using equivalent methods across temperatures (12, 22, and 37°C). The *Arthrobacter* sp. had the highest growth rate at 37°C (0.36 h^-1^), as did the *Pseudomonas* sp. (0.48 h^-1^). The *Paracoccus* isolate had the highest growth rate at 22°C (0.24 h^-1^; **Figure 4**). Growth across a wide range of temperatures has been seen for previous isolates of deep subsurface heterotrophs (Biddle et al., 2005). Additionally, we saw low temperatures induce the production of extra polymeric substances, or EPS, which can be seen in the growth curves that do not plateau (**Figure 4**). This response may be induced *in situ*, which may allow greater survival in an environment such as North Pond, as the development of EPS around a cell can allow for carbon resources to be consumed in the oligotrophic conditions of a sediment core, and also allow for maintained water availability as the deeper sediments are under greater pressure and typically have less pore fluid than shallow ones (Marx et al., 2009).

Given the reasonably high pore water ${NO}_{3}^{-}$ concentrations (up to 35 μM) at 4 and 68 mbsf at U1382B and the very low O_2_ concentration (under 20 μM) at these depths (**Figure 3**, Expedition 336 Scientists, 2012; Orcutt et al., 2013), we tested growth of isolates in anaerobic, heterotrophic, nitrate-reducing media. Also tested was fermentation capability, with the same media as for nitrate-reduction, without nitrate amendment. No growth was observed in either anaerobic media. Our findings are not consistent with the documented nitrate-reducing capabilities of all three isolate genera (Alefounder et al., 1983; Carlson and Ingraham, 1983; Eschbach et al., 2003). An anomalous sharp peak in Mn(II) was measured in porewaters of sediments from just below 50 mbsf at Hole U1382B (**Figure 3**). Given that the *Pseudomonas* and *Paracoccus* isolates originated near the Mn(II) peak at U1382B, we also evaluated our isolates for the capacity to aerobically oxidize Mn(II). Members of the *Actinobacteria* and *Pseudomonas* are known to oxidize Mn(II) (Okazaki et al., 1997; Tebo et al., 2005). Growth was observed from all three isolates, but no Mn-oxides were visually precipitated, indicating a lack of Mn-oxidation capability. These tests were performed after the isolates had been in culture for over a year and thus it is possible that Mn-oxidation capacity was lost as previously reported (Gregory and Staley, 1982).

### Genome Sequencing of Cultivated Bacteria

Isolates genomes were sequenced to validate our physiological data and compare these subsurface isolates to surface relatives. The *Arthrobacter* draft genome assembled into 99 contigs, with a total genome size estimated at 3.9 Mb with a GC content of 65% (**Table 1**). The *Pseudomonas* isolate genome assembled into 24 contigs, with an estimated genome size of 4.7 Mb and a GC content of 60% (**Table 1**). The *Paracoccus* genome assembled into 21 contigs, with an estimated genome size of 3.3 Mb and a GC content of 68% (**Table 1**). All genomes have over 99% completeness, based on the detection of marker genes, and are representative of only one organism. Based on average nucleotide identity (ANI) of equal to or greater than 90% ANI with other genomes, two of these genomes formed cliques with other marine and extreme environment isolates. The *Paracoccus* genome formed a genome clique with *Paracoccus* sp. 361 (Gp0119386) from the Baltic Sea; the *Arthrobacter* formed a clique with *Arthrobacter* sp. 35/47 (Ga0012047) from Antarctic soils. The *Pseudomonas* isolate did not form any cliques, which is typical for this group of microbes, despite the high number of closely related genomes based on ribosomal sequences (25 complete or draft genomes in IMG; Markowitz et al., 2012; database accessed on December 28, 2015).

**FIGURE 5:**
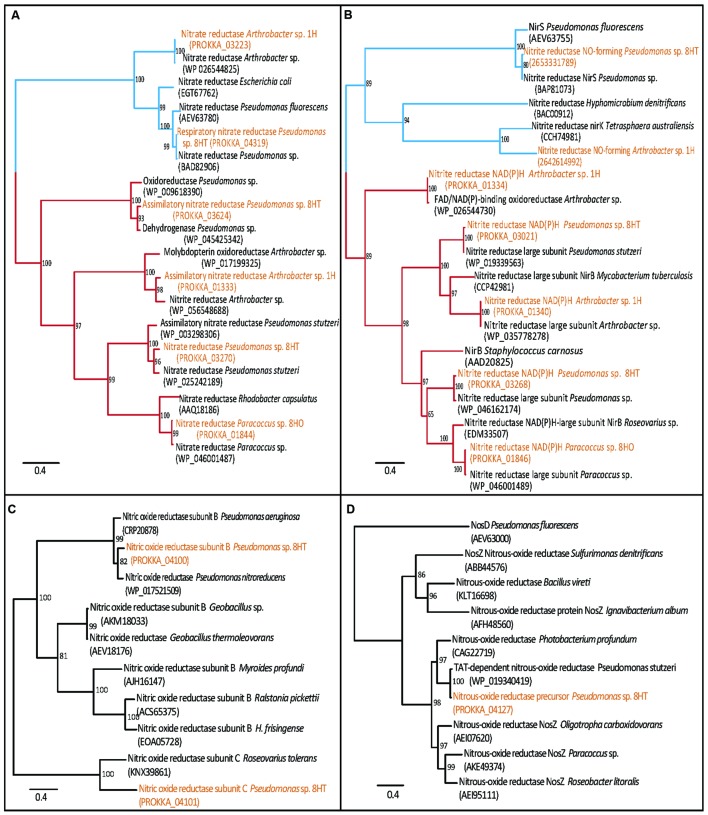
**Maximum-likelihood trees of nitrogen cycle proteins present in genomes, with dissimilatory branches in blue and assimilatory branches in red.** **(A)** Nitrate reductase; showing *Arthrobacter* sp. 1H and *Pseudomonas* sp. 8HT have dissimilatory nitrate reductase, narG, and also assimilatory types (*Pseudomonas* sp. 8HT has two separate types). *Paracoccus* sp. 8HO and *Arthrobacter* sp. 1H each have one type of assimilatory nitrate reductase. **(B)** Nitrite reductase; showing all isolates have assimilatory type nitrite reductases. **(C)** Nitric oxide reductase; only the *Pseudomonas* sp 8HT isolate contains one, subunits **(B)** and **(C)** are shown **(D)** Nitrous oxide reductase; only found in *Pseudomonas* sp. 8HT. Proteins found in the genomes are highlighted in orange. Confidence values are located at the branch nodes.

The *Arthrobacter* sp. 1H isolate from 4 mbsf possessed a denitrifying nitrate reductase gene cluster (Supplementary Table S2; **Figure 5**). Specifically, *narG*, *narH*, *narJ*, and *narI* were all detected, suggesting a capability for the dissimilatory reduction of nitrate (${NO}_{3}^{-}$) to nitrite (NO_2_^-^). This genome also possesses the genes for nitrite reductase (NADH) small and large subunits (*nirD* and *nirB*, respectively), completing the dissimilatory reduction pathway to ammonia. The nitrite reductase NO-forming enzyme (*nirK*) is also encoded within the genome, which performs the second step in the denitrification pathway to nitrogen. The ability to reduce nitrate is not widespread in the *Arthrobacter* genus, with only 8 of the 75 available full to draft genomes available in IMG encoding similar genes, including this North Pond isolate (Markowitz et al., 2012; database accessed on December 28, 2015). Previous detection of nitrate reduction in *Arthrobacter* noted the difficulties of getting the cells to activate the denitrification pathway in culture, and our cultivation setup may not have enabled the shift of the cells into denitrification (Eschbach et al., 2003). Besides the ability of dissimilatory reduction of nitrate, the *Arthrobacter* isolate also encodes genes for the assimilatory nitrate reductase enzymes (the catalytic *nasA* and electron transfer *nasB* subunits). Alcohol dehydrogenase and lactate dehydrogenase were present, but we were unable to map the entire mixed acid fermentation previously proposed for *A. globiformis* (Eschbach et al., 2003). In general, the genome showed a high degree of auxotrophy, unable to biosynthesize 6 amino acids and coenzyme A, which may explain why this organism in particular was the one isolated from the enrichment procedure in rich media. No genes for motility or urease were observed.

The *Paracoccus* sp. 8HO genome contained multiple genes involved with nitrate processing including *nasA, nasE, nasD* and regulation genes such as *nirQ* (Supplementary Table S2). It contains the genes for nitrite reductase (NADH) small and large subunits (*nirD* and *nirB*, respectively) to ammonia. Although *P. denitrificans* is the canonical organism for the study of denitrification in this group and many other *Paracoccus* species can denitrify via the nap pathway, we did not find good genomic evidence of a dissimilatory nitrate reduction pathway, and interpret the nitrate reduction genes seen as playing a mostly assimilatory role (Supplementary Table S2; **Figure 5**; Alefounder et al., 1983). Considering our inability to activate dentrification activity in culture, along with the genomic evidence, we presume this organism is not capable of conserving energy through nitrate reduction. This genome was also auxotrophic for eight amino acids, perhaps explaining its success in complex media cultivation and contained genes for α, β, γ urease subunits and formate dehydrogenase, suggesting that ammonia recycling and potential for anaerobic activity exist. This genome contained 44 genes from KEGG pathways for cell motility, including genes for flagellar assembly and chemotaxis (Che A, B, D, W, X, Y). Motility requires a high level of electron flow for its operation, and while motility has been suggested in the subsurface (Orsi et al., 2013), it is unknown if the system would operate in this organism *in situ*.

The genome of the *Pseudomonas* sp. 8HT isolate from 68 mbsf contained the nitrate-reducing genes seen in *Paracoccus* sp. 8HO, as well as those genes necessary for reduction of NO to N_2_O (*norE*, *norF*, *norC*, *norB*, *norQ*, and *norD*), and N_2_O to N_2_ (*nosR*, *nosZ*, *nosD*, *nosF*, *nosY*, and *nosL*) and periplasmic nitrate reductase genes *napA* and *napB* (Supplementary Table S2; **Figure 5**). *P. stutzeri* is a well-known denitrifying bacterium, and this isolate genome contains the transcriptional regulator, NirQ, suggesting activity should be able to be stimulated in proper growth conditions (Supplementary Table S2; Berks et al., 1995). We anticipate that further lab work would be able to induce the expression of these genes in laboratory culture, but for now, the presented genomic information is the only evidence of potential dissimilatory nitrate reduction. Genes are present for β, γ urease subunits (not the catalytic α subunit), formate dehydrogenase and lactate dehydrogenase, suggesting as with *Paracoccus* that potential for ammonia recycling and anaerobic activity exist. The genome is also auxotrophic for 12 amino acids and coenzyme A biosynthesis, which may be why it grew in complex cultivation media. Over 117 genes were mapped to cellular motility pathways, including multiple copies of chemotaxis proteins and flagellar synthesis, as stated before, it is unknown if these would be used *in situ*, however, they could be advantageous for finding food in a limited physical environment.

We investigated the three genomes for genes known to be involved in manganese oxidation, considering its increase in this environment (**Figure 3**), searching for genes previously implicated in the process (Brouwers et al., 1999; Dick et al., 2006, 2008). There were no genes annotated as *mnxG*, and at first glance, none annotated as *cumA*. However, when we investigated for more general multicopper oxidase annotations, we did find a gene in the *Pseudomonas* isolate genome that was 96% similar to CumA-like oxidase, which is suggested to be the gene responsible for Mn oxidation in *P. putida* (Brouwers et al., 1999). Considering the negative result in the culture screening, we cannot confirm this gene confers the ability to oxidize manganese.

**FIGURE 6:**
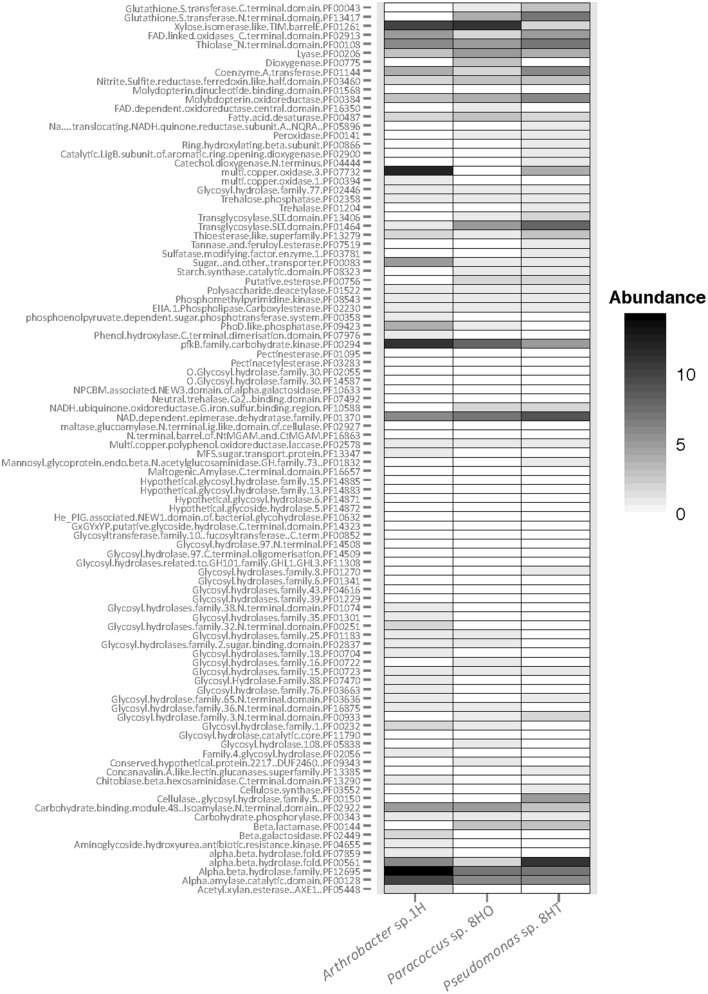
**Abundance of carbohydrate-active enzymes by protein family (pfam) across the three isolate genomes: *Arthrobacter* sp. 1H, *Paracoccus* sp. 8HO, *Pseudomonas* sp. 8HT.** Black indicates more abundant genes, white indicates absence of genes. Categories without matches are not shown.

We examined all three isolates for carbohydrate-active enzymes (CAZy: Cantarel et al., 2009). As anticipated for aerobic heterotrophs, numerous genes were found, including 144 in *Arthobacter* sp. 1H, 140 in *Pseudomonas* sp. 8HT and 113 in *Paracoccus* sp. 8HO (**Figure 6**). The shallow isolate, *Arthrobacter* sp. 1H, contained abundant genes for glycosyl hydrolases and carbon processing compared to the deeper isolates. It had a higher number of α/β hydrolase (PF12695) and α amylase (PF00128) genes compared to the deeper isolates (**Figure 6**). Many genes are shared between all isolates, but only a few categories were present in abundance in the deeper isolates, *Paracoccus* and *Pseudomonas* and not the shallow *Arthrobacter* sp. (**Figure 6**). This includes β-lactamase (PF00144), a putative esterase (PF00756) and glutathione *S*-transferase (GST) in both C and N terminal domains (PF13417, PF00043), which has 10 total genes in *Pseudomonas* sp. 8HT and 5 in *Paracoccus* sp. 8HO, but none in *Arthrobacter* sp. 1H. These genes are noted for conferring the ability to survive oxidative stress (Vuilleumier and Pagni, 2002). We make the presumption that the sediment column is seeded from the overlying water, and when deposited, the *Arthrobacter* sp. 1H has a higher ability to utilize sugars. However, as the sediment column builds on top of it, it and the rest of its phylum will be pushed to lower depths of sediment, where they encounter the low oxygen zone that exists mid-sediment column (**Figure 3**). Our 16S rRNA gene amplicon sequencing of environmental DNA shows that there is a decrease in *Actinobacteria* and an increase in *Proteobacteria* when the sediment is reintroduced to oxygen diffusing up from flow above the basalt layer (**Figure 3**). The *Proteobacteria* have been noted to possess a greater abundance of GSTs than other bacterial phyla (Allocati et al., 2009), which could confer on them a greater ability to recover from the oxidative stress that can be caused from a reintroduction of oxygen after existence in a low or no-oxygen mid sediment column. This, of course, is speculation and should be investigated further using these laboratory cultures or environmental metagenomic data. Overall, we do not see clear indication in a differential ability to utilize carbon sources from these isolates dependent on depth of recovery, but we suggest the ability to withstand oxidative stress may be more crucial in survival downcore at this site.

### Summary

The discrepancy between physiological characterization and genomic data hints at the difficulty of using single cultivation strategies. Given the presence of genes, it is likely that *Arthrobacter* and *Pseudomonas* isolates are capable of nitrate reduction *in situ*. This finding is particularly compelling since the geochemical profiles of their respective isolation depths suggested this metabolic versatility may be important in these low oxygen, nitrate containing porewaters. Recent work has indicated the importance of microbial nitrogen cycling in North Pond sediments (Wankel et al., 2015). In areas of low oxygen, high rates of denitrification were measured. Despite the presumed slow growth rate of organisms in these sediments, isotopic evidence was seen for the energetically costly pathways of nitrate assimilation, nitrification and dentrification (Wankel et al., 2015). The previous work postulates that greater regulation of these processes should be needed in an oligotrophic sedimentary setting such as North Pond. However, our study shows that recoverable isolates are quite similar to species in higher energy environments (**Figures 2**, **3**, and **5**), yet cultivation work suggests that metabolisms beyond aerobic heterotrophy are difficult to induce. The distribution of carbohydrate-active enzymes suggests that genes for oxidative stress may play a role in determining if cells can recover when reintroduced to oxygen in this sediment column (**Figure 6**). With genomes in hand, further investigations can be taken to examine additional metabolic controls and usage of carbon substrates by these isolates, which provide deep subsurface species on which future adaptation or metabolic tests may be performed.

## Conclusion

We present the enrichment and isolation of three species of aerobic heterotrophs from the shallow and deep micro-aerobic zones of the U1382B sediment column. The three isolates were detected in very low relative abundance at their respective sediment depths according to culture-independent molecular analysis. This phenomenon of culturable microbes often being ‘rare’ community members is common across many environments (Shade et al., 2012), including the marine deep biosphere (Teske, 2006). However, these isolates, examined in pure culture and through genomics, have traits such as genes for nitrate reduction and denitrification, that would aid their survival in this environment where models have shown these activities to be important. These isolates can serve as type-species for investigations into adaptations for life in the deep biosphere.

## Author Contributions

JR: performed research, analyzed data, wrote paper. RL-Z: analyzed data, wrote paper. KW: funded research, wrote paper. JB: funded research, analyzed data, wrote paper.

## Conflict of Interest Statement

The authors declare that the research was conducted in the absence of any commercial or financial relationships that could be construed as a potential conflict of interest.

## Footnotes

1 https://www.arb-silva.de/ngs

2 www.arb-silva.de/aligner

3 https://github.com/Victorian-Bioinformatics-Consortium/nesoni.

4 http://blast.ncbi.nlm.nih.gov/Blast.cgi

5 www.ebi.ac.uk/interpro.

6 www.cazy.org
