# Supplementary material for: Deep Subsurface Life from North Pond: Enrichment, Isolation, Characterization and Genomes of Heterotrophic Bacteria
Source: Front Microbiol. 2016 May 10;7:678. doi: 10.3389/fmicb.2016.00678 (PMC4861733; doi:10.3389/fmicb.2016.00678)
Supplement: Supplementary file 1 [file Data_Sheet_1.PDF]

Supplementary Material:

Supplementary Table 1: Enrichments started by J. Russell on IODP Expedition 336 samples

Enrichment for:	IODP Hole	Core section	Depth (mbsf)	Matrix
**Heterotrophs**	U1382B	1H-4	4	Sediment
	U1382B	3H-2	17.7	Sediment
	U1382B	5H-1	34.7	Sediment
	U1382B	7H-6	60.7	Sediment
	U1382B	8H-4	67	Sediment
	U1382B	9H-6	79.5	Sediment
	U1383E	1H-2	2.1	Sediment
	U1383E	3H-4	17.2	Sediment
	U1383E	5H-2	33.2	Sediment
	U1383E	6H-6	47.7	Sediment
	U1384A	2H-6	12.1	Sediment
	U1384A	3H-5	19.8	Sediment
	U1384A	6H-6	50.9	Sediment
	U1384A	9H-7	79.3	Sediment
	U1382A	5R-2	134	Basalt
	U1382A	7R-2	153	Basalt
	U1382A	9R-2	173	Basalt
	U1383C	13R-1	174	Basalt
	U1383C	19R-1	212	Basalt
	U1383C	27R-1	285	Basalt
	U1383C	31R-3	315	Basalt
**Sulfate reduction**	U1382B	4H-4	28.7	Sediment
	U1382B	6H-6	48.7	Sediment
	U1383E	3H-2	14.4	Sediment
	U1383E	4H-3	24	Sediment
	U1384A	4H-6	30.9	Sediment
	U1384A	5H-4	37.4	Sediment
	U1382A	5R-1	132	Basalt
	U1382A	6R-1	142	Basalt
	U1382A	7R-2	153	Basalt
	U1382A	8R-4	163	Basalt
	U1382A	9R-1	172	Basalt
	U1383C	3R-1	77.8	Basalt
	U1383C	24R-1	256	Basalt
**Sulfide oxidation**	U1382A	5R-1	132	Basalt
	U1382A	6R-1	142	Basalt
	U1382A	7R-2	153	Basalt
	U1382A	8R-4	163	Basalt
	U1382A	10R-1	181	Basalt
	U1383C	6R-2	106	Basalt
	U1383C	23R-1	248	Basalt
**Mn reduction**	U1382B	6H-6	48.7	Sediment
	U1382A	6R-1	142	Basalt
**Mn oxidation**	U1382A	3R-4	118	Basalt
	U1382A	7R-2	153	Basalt
	U1383C	9R-3	138	Basalt
	U1383C	20R-2	220	Basalt

Enrichment for:	IODP Hole	Core section	Depth (mbsf)	Matrix
**Methanogenesis**	U1382B	3H-5	22	Sediment
	U1382B	6H-3	46	Sediment
	U1383E	3H-2	14	Sediment
	U1383E	3H-6	20	Sediment
	U1384A	4H-4	28	Sediment
	U1384A	5H-4	37	Sediment
	U1382A	3R-4	118	Basalt
	U1382A	6R-1	142	Basalt
	U1382A	7R-2	153	Basalt
	U1382A	8R-4	163	Basalt
	U1382A	9R-1	172	Basalt
	U1383C	2R-2	71	Basalt
	U1383C	23R-1	248	Basalt
**Iron reduction**	U1382B	9H-3	75	Sediment
	U1382B	9H-6	80	Sediment
	U1384A	7H-5	58	Sediment
	U1384A	9H-7	79	Sediment
	U1383C	9R-3	138	Basalt
	U1383C	14R-2	184	Basalt
	U1383C	20R-2	220	Basalt
	U1383C	30R-3	306	Basalt
**Iron oxidation**	U1384A	7H-5	58	Sediment
	U1384A	9H-7	79	Sediment
	U1382A	3R-4	118	Basalt
	U1382A	7R-2	153	Basalt
	U1383C	2R-2	71	Basalt
	U1383C	14R-2	184	Basalt
	U1383C	20R-2	220	Basalt
	U1383C	30R-3	306	Basalt
	U1383C	31R-3	314	Basalt
**Iron oxidation/**	U1382B	9H-3	75	Sediment
**Nitrate reduction**	U1382B	9H-6	80	Sediment
	U1383E	2H-4	7.8	Sediment
	U1383E	4H-3	24	Sediment
	U1384A	3H-5	19.8	Sediment
	U1384A	5H-4	37.4	Sediment
	U1384A	5H-6	40	Sediment
	U1384A	6H-4	48	Sediment
	U1384A	9H-7	79	Sediment
	U1382A	5R-2	132	Basalt
	U1382A	7R-2	153	Basalt
	U1382A	8R-4	163	Basalt
	U1383C	6R-1	105	Basalt
	U1383C	12R-1	164	Basalt
	U1383C	20R-2	220	Basalt
	U1383C	23R-1	248	Basalt

Supplementary Table 2: Detailed annotation of genes involved in nitrogen pathways

Pseudomonas

Scaffold ID	CDS begin	CDS end	Strand	EC number	Gene ID	Subject ID	Prokka ID	Prokka annotation	NCBI BlastP	InterPro domains
NODE_7	6327	7301	+	1.13.12.16		Q9I4V0	PROKKA_00130	Nitronate monooxygenase	2-nitropropane dioxygenase [Pseudomonas sp. MT-1]	Nitronate monooxygenase (IPR004136).
NODE_7	147983	148321	-		glnK	P0AC55	PROKKA_00260	Nitrogen regulatory protein P-II 2	nitrogen regulatory protein P-II 2 [Proteobacteria]	Nitrogen regulatory protein PII/ATP phosphoribosyltransferase, C-terminal (IP
NODE_3	581791	582273	-		napB	P39186	PROKKA_01504	Periplasmic nitrate reductase%2C electron transfer subunit precursor	cytochrome c [Pseudomonas stutzeri] / nitrate reductase cytochr	Nitrate reductase cytochrome c-type subunit NapB (IPR005591)
NODE_3	584786	585046	-			PRK10553	PROKKA_01506	assembly protein for periplasmic nitrate reductase	glutamate synthase [Pseudomonas]	Chaperone NapD, nitrate reductase assembly (IPR005623)
NODE_3	803937	804503	+	1.-.-.-	ydjA	P0ACY1	PROKKA_01706	Putative NAD(P)H nitroreductase YdjA	nitroreductase [Pseudomonas sp. 10B238]	Putative NAD(P)H nitroreductase YdjA-like (IPR026021)
NODE_2	97327	97791	+	2.7.1.-	ptsN	P69829	PROKKA_01838	Nitrogen regulatory protein	PTS fructose transporter subunit IIA [Pseudomonas stutzeri]	Phosphotransferase system, IIA-like nitrogen-regulatory protein PtsN (IPR006
NODE_2	491795	492838	+	1.13.12.16		Q9I4V0	PROKKA_02195	Nitronate monooxygenase	2-nitropropane dioxygenase [Pseudomonas sp. MT-1]	Nitronate monooxygenase (IPR004136)
NODE_2	833947	834549	-	1.-.-.-	mhqN	P96692	PROKKA_02495	Putative NAD(P)H nitroreductase MhqN	nitroreductase [Pseudomonas stutzeri]	Nitroreductase (IPR029479)
NODE_2	883749	884807	+	1.-.-.-		Q5XCB9	PROKKA_02537	Putative NAD(P)H nitroreductase	nitroreductase [Pseudomonas stutzeri]	Nitroreductase (IPR029479)
NODE_2	1294907	1296343	-		glnG_1	P41789	PROKKA_02932	Nitrogen regulation protein NR(I)	nitrogen regulation protein NR(I) [Pseudomonas sp. 10B238]	Signal transduction response regulator, nitrogen regulation NR(I) (IPR010114)
NODE_2	1296340	1297422	-	2.7.13.3	glnL	P0AFB5	PROKKA_02933	Nitrogen regulation protein NR(II)	sensor histidine kinase [Pseudomonas stutzeri]	regulation of transcription, DNA-templated (Histidine kinase domain (IPR005
NODE_1	62872	65445	+	1.7.1.4	nirB	P42435	PROKKA_03021	Nitrite reductase [NAD(P)H]	nitrite reductase large subunit [Pseudomonas stutzeri]	Nitrite reductase [NAD(P)H] large subunit, NirB (IPR012744)
NODE_1	65445	65822	+	1.7.1.15	nirD	P0A9I8	PROKKA_03022	Nitrite reductase (NADH) small subunit	nitrite reductase small subunit [Pseudomonas sp. 10B238]	Nitrite reductase [NAD(P)H] large subunit, NirD bacterial-type (IPR017881)
NODE_1	316605	317846	+		nrtA	P73452	PROKKA_03256	Nitrate transport protein NrtA precursor	nitrate transporter [Pseudomonas sp. 10B238]	(Periplasmic binding protein-like II)
NODE_1	325915	327126	+		nasA	P42432	PROKKA_03265	Nitrate transporter	MFS transporter [Pseudomonas stutzeri]	Nitrate transporter (IPR004737)
NODE_1	329704	332154	+	1.7.1.4	nasD_2	P42435	PROKKA_03268	Nitrite reductase [NAD(P)H]	nitrite reductase large subunit [Pseudomonas sp. 10B238]	Nitrite reductase [NAD(P)H], large subunit (IPR017121)
NODE_1	332204	332530	+	1.7.1.4	nasE	P42436	PROKKA_03269	Assimilatory nitrite reductase [NAD(P)H] small subunit	nitrite reductase [Pseudomonas stutzeri]	Nitrite reductase [NAD(P)H] small subunit, NirD (IPR012748)
NODE_1	332575	335280	+	1.7.99.4	narB	P39458	PROKKA_03270	Nitrate reductase	nitrate reductase [Pseudomonas stutzeri]	Molybdopterin oxidoreductase (IPR006656)
NODE_1	478122	478940	-		vnfA	P12627	PROKKA_03419	Nitrogen fixation protein VnfA	sigma-54-dependent Fis family transcriptional regulator [Pseudon	Transcriptional regulatory protein FlbD (IPR029995)
NODE_1	478970	479518	-		glnG_2	P41789	PROKKA_03420	Nitrogen regulation protein NR(I)	sigma-54-dependent Fis family transcriptional regulator [Pseudon	Transcriptional regulatory protein FlbD (IPR029995)
NODE_1	480841	482310	-		ntrC	P10577	PROKKA_03422	Nitrogen assimilation regulatory protein	MULTISPECIES/ sigma-54-dependent Fis family transcriptional reg	CheY-like superfamily (IPR011006) RNA polymerase sigma factor 54 interactio
NODE_1	483598	484815	-		nrfG	Q8X5S3	PROKKA_03425	Formate-dependent nitrite reductase complex subunit NrfG	c-type cytochrome biogenesis protein CcmI [Pseudomonas stutze	Cytochrome c-type biogenesis protein CcmI (IPR017560) Tetratricopeptide rep
NODE_1	699561	701657	-	1.7.99.4	nasC	P42434	PROKKA_03624	Assimilatory nitrate reductase catalytic subunit	dehydrogenase [Pseudomonas sp. MT-1]	Molybdopterin oxidoreductase (IPR006656)
NODE_1	1186445	1188016	-		norR2	Q9K4U8	PROKKA_04094	Nitric oxide reductase transcription regulator NorR2	nitric oxide reductase transcription regulator [Pseudomonas stutz	GAF domain (IPR003018) RNA polymerase sigma factor 54 interaction domain
NODE_1	1192878	1194305	-	1.7.2.5	norB	P98008	PROKKA_04100	Nitric oxide reductase subunit B	nitric oxide reductase [Pseudomonas sp. MT-1]	Cytochrome c oxidase subunit I (IPR000883)
NODE_1	1194309	1194749	-		norC	Q52527	PROKKA_04101	Nitric oxide reductase subunit C	cytochrome c [Pseudomonas stutzeri]/nitric-oxide reductase subu	Cytochrome c-like domain (IPR009056)
NODE_1	1196034	1196840	-		nirQ	Q02441	PROKKA_04104	Denitrification regulatory protein NirQ	ATPase AAA [Pseudomonas sp. MT-1]/denitrification regulatory p	ATPase, dynein-related, AAA domain (IPR011704) CbbQ/NirQ/NorQ, C-termin
NODE_1	1197195	1198904	+	1.7.2.1	nirS_1	P24474	PROKKA_04105	Nitrite reductase precursor	nitrite reductase [Pseudomonas sp. MT-1]	Cytochrome cd1-nitrite reductase-like, haem d1 domain (IPR011048)
NODE_1	1205036	1206529	+	1.7.2.1	nirS_2	P72181	PROKKA_04115	Nitrite reductase precursor	cytochrome CBB3 [Pseudomonas stutzeri]/Nitrite reductase precu	Cytochrome cd1-nitrite reductase-like, haem d1 domain (IPR011048)
NODE_1	1216341	1217648	-			PF05048.7	PROKKA_04126	Periplasmic copper-binding protein (NosD)	copper-binding protein [Pseudomonas stutzeri]/NosD protein [Ps	Nitrous oxide reductase family maturation protein NosD (IPR026464
NODE_1	1217714	1219615	-	1.7.2.4	nosZ	P19573	PROKKA_04127	Nitrous-oxide reductase precursor	TAT-dependent nitrous-oxide reductase [Pseudomonas stutzeri]/r	Nitrous-oxide reductase (IPR023644)
NODE_1	1434313	1435110	-	1.7.99.4	narI	P11350	PROKKA_04315	Respiratory nitrate reductase 1 gamma chain	nitrate reductase [Pseudomonas sp. 10B238]/respiratory nitrate r	Nitrate reductase, gamma subunit (IPR003816)
NODE_1	1435103	1435858	-		narJ	P0AF26	PROKKA_04316	Nitrate reductase molybdenum cofactor assembly chaperone NarJ	nitrate reductase molybdenum cofactor assembly chaperone Nar	Nitrate reductase chaperone, NarJ (IPR003765)
NODE_1	1435862	1437400	-	1.7.99.4	narH	P11349	PROKKA_04317	Respiratory nitrate reductase 1 beta chain	nitrate reductase [Pseudomonas sp. MT-1]/MULTISPECIES: respira	Nitrate reductase, beta subunit (IPR006547)
NODE_1	1437500	1441255	-	1.7.99.4	narG	P09152	PROKKA_04319	Respiratory nitrate reductase 1 alpha chain	nitrate reductase [Pseudomonas sp. MT-1]/membrane-bound nitr	Nitrate reductase, alpha subunit (IPR006468)
NODE_1	1441358	1442845	-		narT	O33854	PROKKA_04320	putative nitrate transporter NarT	MFS transporter [Pseudomonas sp. MT-1]/Putative nitrate transp	Major facilitator superfamily (IPR011701)
NODE_1	1443334	1444935	-		narK	P10903	PROKKA_04322	Nitrate/nitrite transporter NarK	antiporter [Pseudomonas sp. 10B238]/nitrate transporter [Pseud	Major facilitator superfamily domain (IPR020846)
NODE_1	1445234	1447117	+	2.7.13.3	narX	P0AFA2	PROKKA_04323	Nitrate/nitrite sensor protein NarX	sensor protein [Pseudomonas sp. MT-1]/ MULTISPECIES: nitrate/r	Signal transduction histidine kinase, nitrate/nitrite-sensing (IPR016380)
NODE_1	1447114	1447770	+		narL	P0AF28	PROKKA_04324	Nitrate/nitrite response regulator protein NarL	nitrate-dependent two-component reguratory system transcription factor NarL [Pseudomonas sp. MT-1]	
NODE_1	1214019	1214570	-			PF05573.6	PROKKA_04123	NosL	NosL protein [Pseudomonas sp. 10B238]/itrous oxide reduction p	Nitrous oxide reductase accessory protein NosL (IPR008719)
Ga0111029_122	582285	584789	-	1.7.99.4	napA			Nitrate reductase NapA	nitrate reductase catalytic subunit [Pseudomonas sp. MT-1]	Molybdopterin oxidoreductase (IPR006656)

Arthrobacter

Scaffold ID	CDS begin	CDS end	Strand	EC number	Gene ID	Subject ID	Prokka ID	Prokka annotation	NCBI BlastP	InterPro domains
NODE_1	451740	452978	-			PRK10935	PROKKA_00426	nitrate/nitrite sensor protein NarQ	histidine kinase [Arthrobacter sp. 35/47]	Phage shock protein, PspC, N-terminal (IPR007168) Histidine kinase-like ATPas
NODE_3	218611	220005	+		narU	P37758	PROKKA_01332	Nitrate/nitrite transporter NarU	nitrate transporter [Arthrobacter sp. Soil782]	Major facilitator superfamily (IPR011701)
NODE_3	220067	222148	+	1.7.99.4	nasC	P42434	PROKKA_01333	Assimilatory nitrate reductase catalytic subunit	nitrite reductase [Arthrobacter sp. Soil782]/	Molybdopterin oxidoreductase (IPR006656)
NODE_3	222145	223731	+	1.7.1.4	nasD_1	P42435	PROKKA_01334	Nitrite reductase [NAD(P)H]	FAD/NAD(P)-binding oxidoreductase [Arthrobacter sp. 35/47]/fer	FAD/NAD(P)-binding domain (IPR023753) BFD-like [2Fe-2S]-binding domain (I
NODE_3	229049	229426	-	1.7.1.15	nirD	P0A9I8	PROKKA_01339	Nitrite reductase (NADH) small subunit	hypothetical protein [Arthrobacter sp. 35/47]/nitrite reductase sr	Nitrite reductase [NAD(P)H] large subunit, NirD bacterial-type (IPR017881)
NODE_3	229690	232317	+	1.7.1.4	nirB	P42435	PROKKA_01340	Nitrite reductase [NAD(P)H]	nitrite reductase large subunit [Arthrobacter sp. 35/47]	Nitrite reductase [NAD(P)H] large subunit, NirB (IPR012744)
NODE_4	125796	126803	+			PF03060.9	PROKKA_01470	Nitronate monooxygenase	2-nitropropane dioxygenase [Arthrobacter sp. 35/47]	Nitronate monooxygenase (IPR004136)
NODE_6	24534	24872	-		glnB	P64249	PROKKA_01790	Nitrogen regulatory protein P-II	nitrogen regulatory protein P-II 1 [Arthrobacter sp. 35/47]	Nitrogen regulatory protein PII (IPR002187)
NODE_23	6219	9935	+	1.7.99.4	narG	O06559	PROKKA_03223	Nitrate reductase alpha subunit	nitrate reductase [Arthrobacter sp. 35/47]/nitrate reductase A sul	Nitrate reductase, alpha subunit (IPR006468)
NODE_23	9935	11605	+	1.7.99.4	narY	P19318	PROKKA_03224	Respiratory nitrate reductase 2 beta chain	nitrate reductase [Arthrobacter sp. Soil782]/nitrate reductase sub	Nitrate reductase, beta subunit (IPR006547)
NODE_23	11602	12276	+	1.7.99.4	narX_1	P71994	PROKKA_03225	Nitrate reductase-like protein NarX	nitrate reductase [Arthrobacter sp. 35/47]/nitrate reductase moly	Nitrate reductase chaperone, NarJ (IPR003765)
NODE_23	12279	13031	+	1.7.99.4	narX_2	P71994	PROKKA_03226	Nitrate reductase-like protein NarX	nitrate reductase [Arthrobacter sp. 35/47]/nitrate reductase subu	Nitrate reductase, gamma subunit (IPR003816)
NODE_23	13056	14297	+		narK2	P71995	PROKKA_03227	putative nitrate/nitrite transporter NarK2	MFS transporter [Arthrobacter sp. Soil782]/nitrate/nitrite transpc	Major facilitator superfamily (IPR011701)
NODE_23	16116	18740	+	1.7.2.1	aniA	Q02219	PROKKA_03230	Copper-containing nitrite reductase precursor	multicopper oxidase [Arthrobacter sp. 35/47]/putative nitrite red	Nitrite reductase, copper-type (IPR001287)
Ga0073312_104	220330	222411	+	1.7.99.4	nasA			Assimilatory nitrate reductase catalytic subunit	nitrite reductase [Arthrobacter sp. Soil782]	Molybdopterin oxidoreductase (IPR006656) Aspartate decarboxylase-like dom
Ga0073312_105	222408	223994	+	1.7.99.5	nasB			Assimilatory nitrate reductase electron transfer subunit	FAD/NAD(P)-binding oxidoreductase [Arthrobacter sp. 35/47]	FAD/NAD(P)-binding domain (IPR023753)BFD-like [2Fe-2S]-binding domain (IF
Ga0073312_106	154593	157217	-	1.7.2.1	nirK			Nitrite reductase (NO-forming)	multicopper oxidase [Arthrobacter sp. 35/47]	Cupredoxin (IPR008972) Multicopper oxidase, type 3 (IPR011707)

Paracoccus

Scaffold ID	CDS begin	CDS end	Strand	EC number	Gene ID	Subject ID	Prokka ID	Prokka annotation	NCBI BlastP	InterPro domains
NODE_13	152732	153700	-		nirQ	Q51481	PROKKA_00405	Denitrification regulatory protein NirQ	ATPase AAA [Paracoccus sp. 361]/ATPase associated with various	ATPase chaperone, AAA-type, MoxR, predicted (IPR016366)
NODE_11	56028	56450	+	2.7.1.-	ptsN	P69829	PROKKA_00697	Nitrogen regulatory protein	PTS lactose transporter subunit IIC [Paracoccus sp. PAMC 22219]	PTS EIIA type-2 domain (IPR002178)
NODE_11	147434	148663	-			PRK10370	PROKKA_00794	formate-dependent nitrite reductase complex subunit NrfG	c-type cytochrome biogenesis protein CcmI [Paracoccus sp. 361]	Cytochrome c-type biogenesis protein CcmI (IPR017560) Tetratricopeptide-like
NODE_9	123516	124484	-			PF03060.9	PROKKA_01173	Nitronate monooxygenase	2-nitropropane dioxygenase [Paracoccus sp. S4493]	Nitronate monooxygenase (IPR004136).
NODE_17	82835	83605	+		fixK	P29286	PROKKA_01396	Nitrogen fixation regulation protein FixK	transcriptional regulator [Paracoccus sp. S4493]/CRP/FNR family t	RmlC-like jelly roll fold (IPR014710) Cyclic nucleotide-binding-like (IPR018490)
NODE_10	118450	121038	-	1.7.99.4	nasA	Q06457	PROKKA_01844	Nitrate reductase	nitrate reductase [Paracoccus sp. 361]/NasA [Rhodobacter capsul	Molybdopterin oxidoreductase (IPR006656) Molybdopterin oxidoreductase, n
NODE_10	121035	121361	-	1.7.1.4	nasE	P42436	PROKKA_01845	Assimilatory nitrite reductase [NAD(P)H] small subunit	tRNA-(guanine-N1)-methyltransferase [Paracoccus sp. S4493]/Nir	Nitrite reductase [NAD(P)H] small subunit, NirD (IPR012748)
NODE_10	121372	123801	-	1.7.1.4	nasD	P42435	PROKKA_01846	Nitrite reductase [NAD(P)H]	nitrite reductase large subunit [Paracoccus sp. 361]	Nitrite reductase [NAD(P)H], large subunit (IPR017121)
NODE_10	125505	126809	-		nrtA_1	P73452	PROKKA_01849	Nitrate transport protein NrtA precursor	bicarbonate-binding protein [Paracoccus sp. 361]/NasF [Rhodoba	Twin-arginine translocation pathway, signal sequence (IPR006311)
NODE_10	127077	128249	-		nrtA_2	P73452	PROKKA_01850	Nitrate transport protein NrtA precursor	nitrate transporter [Paracoccus sp. 361]/NasS [Rhodobacter capsulatus]	
NODE_6	27108	28220	+	2.7.13.3	glnL	P0AFB5	PROKKA_02375	Nitrogen regulation protein NR(II)	MULTISPECIES: PAS domain-containing sensor histidine kinase [Pa	PAS domain (IPR000014) Signal transduction histidine kinase, dimerisation/ph
NODE_6	28233	29678	+		ntrC	P10577	PROKKA_02376	Nitrogen assimilation regulatory protein	sigma-54-dependent Fis family transcriptional regulator [Paracocc	CheY-like superfamily (IPR011006) P-loop containing nucleoside triphosphate
NODE_6	32010	33401	+		glnG	P41789	PROKKA_02378	Nitrogen regulation protein NR(I)	sigma-54-dependent Fis family transcriptional regulator [Paracocc	CheY-like superfamily (IPR011006) P-loop containing nucleoside triphosphate
NODE_6	233733	234305	-	1.-.-.-	ydjA	P0ACY1	PROKKA_02578	Putative NAD(P)H nitroreductase YdjA	nitroreductase [Paracoccus sp. S4493]	Putative NAD(P)H nitroreductase YdjA-like (IPR026021) This group consists of
NODE_5	119520	119858	-		glnB	P0A9Z1	PROKKA_02886	Nitrogen regulatory protein P-II 1	MULTISPECIES: nitrogen regulatory protein P-II 1 [Paracoccus]	Nitrogen regulatory protein PII (IPR002187) n Gram-negative bacteria, the act
Ga0111030_113	121035	121361		1.7.1.15	nirD			nitrite reductase (NADH) small subunit	tRNA-(guanine-N1)-methyltransferase [Paracoccus sp. S4493]/Nir	Nitrite reductase [NAD(P)H] small subunit, NirD (IPR012748)
Ga0111030_114	121372	123801		1.7.1.16	nirB			nitrite reductase (NADH) large subunit	nitrite reductase large subunit [Paracoccus sp. 361]	Nitrite reductase [NAD(P)H] large subunit, NirB (IPR012744)
